# Benefits of tolvaptan on early dyspnea relief in patients with acute heart failure: A meta‐analysis

**DOI:** 10.1002/clc.23889

**Published:** 2022-08-02

**Authors:** Wenli Shang, Yingying Zhang, Dong Han

**Affiliations:** ^1^ Department of Respiratory and Critical Care Medicine Shaanxi Provincial People's Hospital Xi'an Shaanxi China; ^2^ Department of Respiratory and Critical Care Medicine The Second Affiliated Hospital of Xi'an Jiaotong University Xi'an Shaanxi China

**Keywords:** acute heart failure, early dyspnea relief, meta‐analysis, tolvaptan, worsening renal function

## Abstract

Considering the prevalence of dyspnea in acute heart failure (AHF), its reduction is important to both patients and caregivers. This meta‐analysis was performed to determine the efficacy and safety of tolvaptan on early dyspnea relief in patients with AHF. A systematic search was made of PubMed, Embase, Web of Science, Cochrane Library, and clinicaltrials.gov, without language restrictions. Randomized controlled trials (RCTs) on treatment of AHF with tolvaptan, compared with placebo or blank, were reviewed. Studies were pooled to relative risk (RR), with 95% confidence interval (CI). Five RCTs (enrolling 4857 participants) met the inclusion criteria. Tolvaptan presented significant effects on 12 h dyspnea relief (RR: 1.98; 95% CI: 1.24−3.15; *p *= .004), 24 h/day 1 dyspnea relief (RR: 1.15; 95% CI: 1.07−1.24; *p *= .0003), 48 h dyspnea relief (RR: 1.20; 95% CI: 1.06−1.36; *p *= .004), and 72 h dyspnea relief (RR: 1.18; 95% CI: 1.02−1.37; *p *= .03). No significant increase was noticed in the incidence of worsening renal function in tolvaptan group (RR: 1.10; 95% CI: 0.87−1.39; *p *= .43). Tolvaptan treatment significantly improved patient‐assessed dyspnea early and persistently in patients with AHF.

## 1 INTRODUCTION

Heart failure (HF) is a major international public health problem, which is associated with significant medical and economic challenges. Elevated ventricular (i.e., congestion) filling pressures are the primary reasons for hospitalization in patients with HF.^1^ In the condition of acute heart failure (AHF), congestion leads to worsening symptoms (representatively dyspnea) and contributes to end‐organ dysfunction.^2^ The sensation of dyspnea, or breathlessness, is practically universal in patients with AHF.^3^ Relief from this symptom compels patients to seek medical care. Considering the prevalence of dyspnea in AHF, its reduction is important to both patients and caregivers, and its role in regulatory approval has resulted in this symptom being targeted in clinical trials.^4^, ^5^, ^6^, ^7^

The oral vasopressin‐2 receptor antagonist tolvaptan inhibits the effects of antidiuretic hormone and leads to the excretion of free water in patients with HF.^8^ Although the large‐scale EVEREST (Efficacy of Vasopressin Antagonist in Heart Failure Outcome Study with Tolvaptan) study did not show benefit of tolvaptan over placebo in terms of long‐term clinical outcomes.^9^ Tolvaptan demonstrated statistically significant improvement in patient‐assessed dyspnea at day 1 in both short‐term trials.^5^, ^10^ Meanwhile, the AQUAMARINE (Answering the Question of Tolvaptan's Efficacy for Patients with Acute Decompensated Heart Failure and Renal Failure) trial revealed an improvement in patient‐assessed dyspnea early to 12 h after first dose administration of tolvaptan.^7^ And this effect of dyspnea reduction persists up to 48 h after first dose of tolvaptan. However, dyspnea relief was not statistically different between patients randomized to tolvaptan or placebo at 8, 24, or 48 h after first dose of tolvaptan in another two trials.^4^, ^6^

The aim of the present study therefore was to perform a meta‐analysis of randomized controlled trials (RCTs) to evaluate the efficacy of tolvaptan on early dyspnea relief in patients with AHF.

## 2 METHODS

### 2.1 Data sources and search strategy

This meta‐analysis was designed according to the Preferred Reporting Items for Systematic Reviews and Meta‐Analyses (PRISMA) statement.^11^ The PubMed, Embase, Web of Science, and Cochrane Library, as well as clinicaltrials.gov were searched for studies published up to July 2021.

### 2.2 Study selection

To be eligible for inclusion in the meta‐analysis studies had to meet the following criteria: (a) inclusion of patients aged over 18 years with AHF and dyspnea, and at least one additional sign or symptom of congestion (orthopnea, edema, jugular venous distention, ascites, pleural effusion, rales, or congestion on chest radiograph); (b) use of a randomized controlled design to make a comparison of tolvaptan with placebo or blank; and (c) information of early dyspnea relief by 7‐point Likert scale. Studies were excluded if they included patients with the following: (a) hypotension (systolic blood pressure <90 mmHg); (b) severe renal dysfunction (serum creatinine >3.5 mg/dl or requiring renal replacement therapy); and (c) acute coronary syndrome on admission. The search strings used for the databases were “tolvaptan” AND “heart failure.” The reference lists of any relevant review articles were also screened to identify studies that were potentially been missed in this search. Our study selection process did not apply any language restrictions.

### 2.3 Data extraction and quality assessment

Two reviewers independently screened articles according to the inclusion criteria. The reviewers compared selected studies and differences were resolved by consensus. Data tables were used to collect all relevant data from texts, tables, and figures of each included trial, including author, year of publication, patient number and age, regimens and doses, time from admission to first dose of tolvaptan, history of atrial fibrillation, baseline medication use, heart rate, left ventricular ejection fraction, blood urea nitrogen, systolic blood pressure and serum creatinine, and outcomes such as dyspnea relief at 8, 12, 24 h/day 1, 48, and 72 h, and the incidence of worsening renal function (WRF) (defined as an increase in serum creatinine of ≥0.3 mg/dl). Study quality was assessed using the Detsky Quality Assessment Scale.^12^, ^13^, ^14^, ^15^ This is a 20‐point scale for studies with statistically significant results and a 21‐point scale for studies without statistically significant results.

### 2.4 Data synthesis and statistical analysis

Meta‐analyses were conducted where applicable; otherwise, outcomes were presented in narrative form. Data were analyzed using the RevMan Version 5.4.1. Next, relative risk (RR) for dichotomous outcomes with corresponding 95% confidence intervals (CIs) were computed for individual trials. *χ* ^2^ and Higgins *I* ^2^ tests were used to assess heterogeneity among the included studies. If significant heterogeneity (*p* ≤ .10 for *χ* ^2^ test results or *I* ^2^ ≥ 50%) was obtained, we used a random‐effects model, otherwise a fixed‐effects model was used. And a *p *< .05 was taken to indicate statistical significance.

To assess the robustness of the results, meta‐regression analyses (STATA version 12.0) were carried out for sensitivity analysis^13^, ^16^, ^17^ to test the influence of potential effect modifiers such as simple size, sex, and Detsky quality score. The *p* value of Egger's linear regression test (STATA 12.0) was used to assess the presence of publication bias in included articles for each outcome.

## 3 RESULTS

### 3.1 Study selection and characteristics

**Figure 1:**
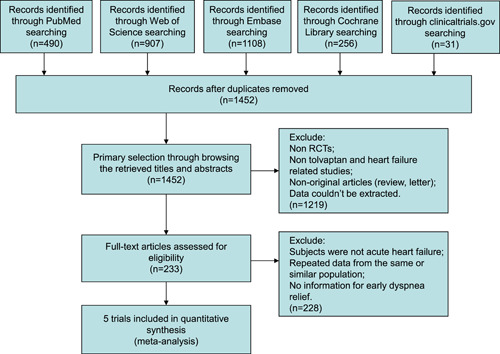
Flow chart for selection of studies. RCTs, randomized controlled trials.

Of 2792 articles recognized by the initial search, 233 were retrieved for more detailed assessment, and five trials in 4 articles^4^, ^5^, ^6^, ^7^ were included in the meta‐analysis (Figure 1). Baseline characteristics of trials included in the meta‐analysis are shown in Table 1. A total of 4857 patients were included: 2431 assigned to tolvaptan treatment groups and 2426 to control groups.

**Table 1:** Baseline characteristics of trials included in meta‐analysis

Study (Ref. #)	Year	Quality score	Time from admission to first dose	Regimen	*n*	Age (years) (SD)	Male (%)	LVEF (%)	Heart rate, beats/min, (SD)
Felker (4)	2017	17	within 24 h	Tolvaptan 30 mg/day placebo	129	66 (13)	66	34 (17)	79 (14)
					128	63 (16)	67	32 (17)	82 (16)
Gheorghiade Trial A (5)	2007	21	within 48 h	Tolvaptan 30 mg/day placebo	1018	65.8 (11.7)	74.0	27.2 (8.2)	79.5 (15.2)
					1030	65.6 (11.9)	76.1	27.3 (8.3)	79.6 (15.4)
Gheorghiade Trial B (5)	2007	21	within 48 h	Tolvaptan 30 mg/day placebo	1054	66.0 (11.7)	72.8	27.8 (7.7)	80.3 (15.9)
					1031	65.6 (12.2)	74.8	27.7 (8.1)	80.0 (16.1)
Konstam (6)	2017	18	within 36 h	Tolvaptan 30 mg/day placebo	122	70 (11)	75.4	35 (16)	NR
					128	67 (13)	72.7	33 (17)	NR
Matsue (7)	2016	19	within 6 h	Tolvaptan 15 mg/day blank	108	72.99 (8.90)	66.7	45.4 (18.1)	94.2 (27.3)
					109	72.95 (10.24)	63.3	46.8 (16.4)	88.6 (23.4)

SBP, mm Hg (SD)	BUN, mg/dl (SD)	Serum creatinine, mg/dl (SD)	Diabetes (%)	History of atrial fibrillation (%)	ACEI or ARB (%)	Beta‐blocker (%)	Aldosterone antagonist (%)
119 (21)	32 (18)	1.48 (0.70)	54	47	62	92	33
117 (19)	31 (17)	1.44 (0.60)	55	56	60	88	31
120.1 (19.9)	29.5 (15.1)	1.3 (0.5)	40.2	41.4	83.8	69.6	55.9
119.4 (18.8)	29.8 (16.2)	1.4 (0.5)	39.6	41.7	84.2	70.0	56.4
121.5 (19.9)	30.3 (16.4)	1.4 (0.5)	39.4	45.6	84.7	72.0	51.7
120.9 (20.0)	31.0 (17.5)	1.4 (0.7)	35.6	44.6	84.0	69.3	53.1
122 (22)	38.5 (17.5)	1.7 (0.5)	49.2	36.1	49.2	75.4	30.3
122 (20)	36.9 (18.5)	1.7 (0.6)	46.9	28.9	40.6	71.9	32.0
145.8 (32.9)	28.0 (NR)	1.59 (0.05)	38.9	55.6	41.7	38.0	17.6
142.1 (28.1)	25.0 (NR)	1.59 (0.04)	49.5	50.5	37.6	39.4	24.8

Abbreviations: ACEI, angiotensin‐converting enzyme inhibitor; ARB, angiotensin receptor blocker; BUN, blood urea nitrogen; LVEF, left ventricular ejection fraction; NR, not reported; SBP, systolic blood pressure.

### 3.2 8 h dyspnea relief

**Figure 2:**
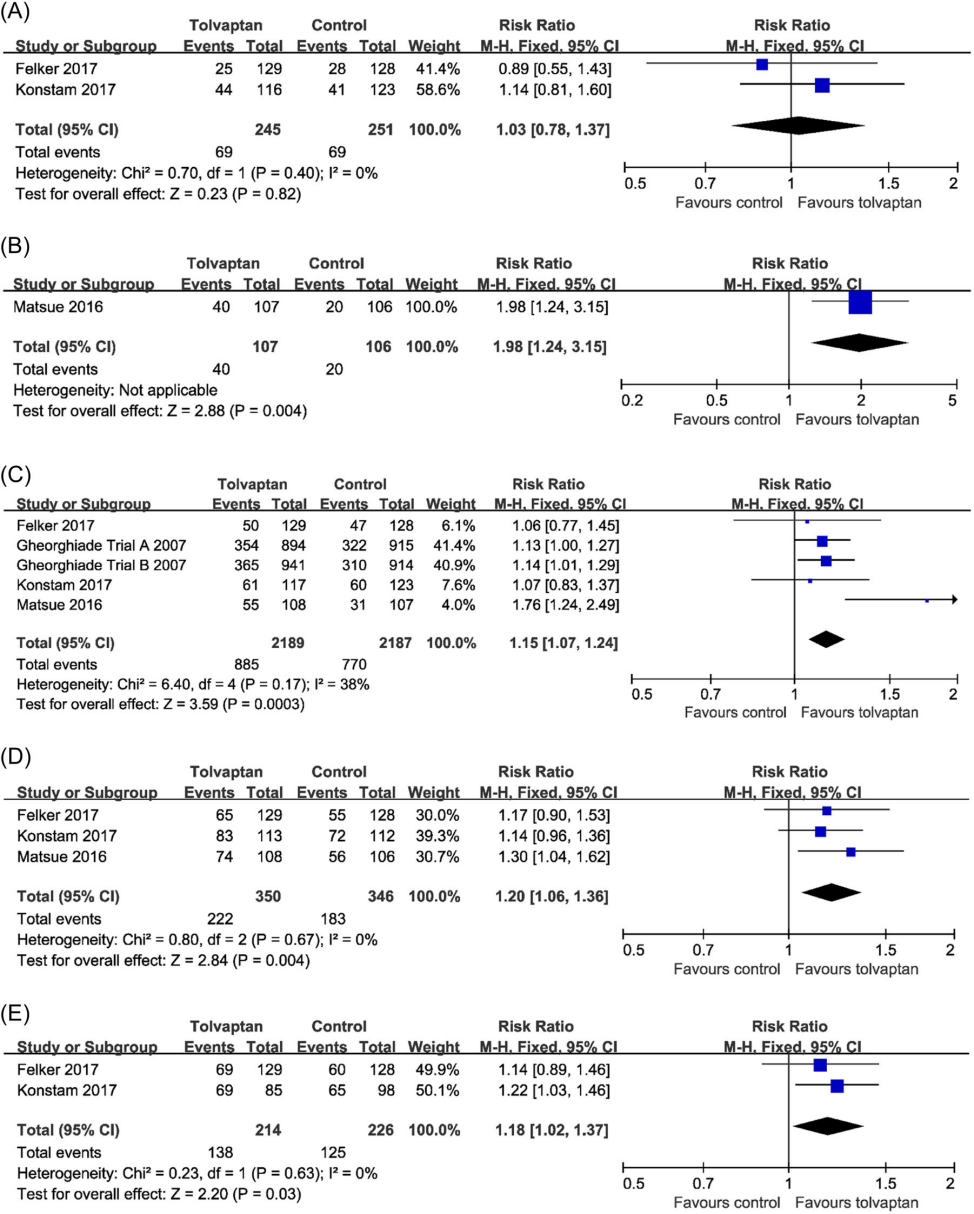
Effects of tolvaptan on patient‐assessed dyspnea at (A) 8, (B) 12, (C) 24 h or day 1, (D) 48, and (E) 72 h. CI, confidence interval, M−H, Mantel−Haenszel.

Data from two trials (496 patients) showed that, dyspnea relief by 7‐point Likert scale was similar between groups at 8 h (28.2% moderately or markedly improved with tolvaptan vs. 27.5% control; RR: 1.03; 95% CI: 0.78−1.37; *p* = .82) after first dose of tolvaptan. There was no significant heterogeneity (*I* ^2^ = 0%; *p* = .40) (Figure 2A).

### 3.3 12 h dyspnea relief

Data from one trial (213 patients) showed that, dyspnea reduction was greater with tolvaptan compared with the control conditions at 12 h (37.4% moderately or markedly improved with tolvaptan vs. 18.9% control; RR: 1.98; 95% CI: 1.24−3.15; *p* = .004) (Figure 2B).

### 3.4 24 h/day 1 dyspnea relief

Data on dyspnea relief at 24 h or day 1 were available from five randomized trials (4376 patients). Compared with the control conditions, tolvaptan significantly improved patient‐assessed dyspnea (40.4% moderately or markedly improved with tolvaptan vs. 35.2% control; RR: 1.15; 95% CI: 1.07−1.24; *p* = .0003). There was no significant heterogeneity (*I* ^2^ = 38%; *p* = .17) (Figure 2C). Egger's test (*p* = .497) did not show evidence of publication bias.

### 3.5 48 h dyspnea relief

The dyspnea reduction at 48 h were evaluated in three studies (696 patients). Compared with the control conditions, dyspnea reduction was greater with tolvaptan at 48 h (63.4% moderately or markedly improved with tolvaptan vs. 52.9% control; RR: 1.20; 95% CI: 1.06−1.36; *p* = .004). There was no significant heterogeneity (*I* ^2^ = 0%; *p* = .67) (Figure 2D). Egger's test (*p* = .738) did not show evidence of publication bias.

### 3.6 72 h dyspnea relief

Data on dyspnea reduction at 72 h were available from two randomized trials (440 patients). Tolvaptan treatment led to a significant dyspnea relief compared with the control conditions at 72 h (64.5% moderately or markedly improved with tolvaptan vs. 55.3% control; RR: 1.18; 95% CI: 1.02−1.37; *p* = .03). There was no significant heterogeneity (*I* ^2^ = 0%; *p* = .63) (Figure 2E).

### 3.7 WRF

**Figure 3:**
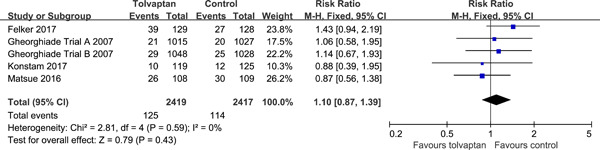
Effects of tolvaptan on the incidence of worsening renal function.

Data on the incidence of WRF were available from five randomized trials (4836 patients). There were no significant differences between groups in the incidence of WRF (5.2% with tolvaptan vs. 4.7% control; RR: 1.10; 95% CI: 0.87−1.39; *p* = .43). There was no significant heterogeneity (*I* ^2^ = 0%; *p* = .59) (Figure 3). Egger's test (*p* = .469) did not show evidence of publication bias.

### 3.8 Sensitivity analysis

Our results were mostly confirmed when potential effect modifiers were introduced as covariates in the meta‐regression analysis. In this analysis, no significant impact was found on either 24 h/day 1 dyspnea relief, 48 h dyspnea relief, or WRF (Table 2).

**Table 2:** Potential effect modifier with change in *τ* ^2^ nd statistical significance for each outcome

	Change in *τ* ^2^	*p* Value
24 h/day 1 dyspnea relief		
Detsky quality score	−0.02	.984
Sample size	−0.42	.706
Men	−1.14	.335
48 h dyspnea relief		
Detsky quality score	0.65	.632
Sample size	−0.41	.750
Men	−0.73	.600
Worsening renal function		
Detsky quality score	−0.70	.532
Sample size	0.07	.949
Men	−0.16	.880

## 4 DISCUSSION

To our knowledge, this meta‐analysis is the first designed specifically to assess RCTs that have explored the effect of tolvaptan treatment on early dyspnea relief in patients with AHF. Based on the present results, we observed that the addition of tolvaptan to conventional therapy with loop diuretics result in greater dyspnea improvement in patients hospitalized with AHF, dyspnea, and congestion. The use of tolvaptan significantly improved patient‐assessed dyspnea early to 12 h, and persisted up to 72 h after first dose of tolvaptan. This meta‐analysis differs from those of most other meta‐analyses,^18^, ^19^, ^20^ which mixing different time points and methods to discuss dyspnea relief in patients with HF.

Patient reported outcomes (PRO) are defined as “any report of the status of a patient's health condition that comes directly from the patient, without interpretation of the patient's response by anyone else.”^21^ As an assessment of patients' experiences, PRO are key measurements in patient centered studies. Dyspnea, or the sensation of breathlessness, is one of the most usually assessed PRO's in AHF clinical trials. The sensation of difficulty breathing or shortness of breath prompts patients with AHF to seek medical care.^22^ Early and persistent relief of dyspnea has been associated with improved outcomes.^23^, ^24^ As such, dyspnea reduction is important to both patients and clinicians, particularly with the current focus on patient centered outcomes.

The end points of this meta‐analysis were moderate or marked improvement of dyspnea from baseline according to patient‐reported 7‐point Likert scale (markedly better, moderately better, minimally better, no change, minimally worse, moderately worse, and markedly worse) measured at 8, 12, 24 h/day 1, 48, and 72 h after first dose of tolvaptan. The Likert scales have been the most widely used and accepted measures of dyspnea in AHF patients.^25^ Likert scales include 3‐, 5‐, or 7‐point scales that request patients to evaluate their grade of improvement in reply to therapy on a categorical spectrum ranging from markedly better to markedly worse or an adequate change. The Likert scales have been accepted in multiple AHF clinical studies, as being effective and reliable means capable of discriminating the level of a patient's dyspnea.^26^ After all, symptom relief is at the heart of the problem from a patient's perspective.

The WRF during therapy for AHF has grown more complex in the past few years as it has been accepted that WRF is a very heterogeneous phenomenon and that the prognostic implications may be influenced by whether the WRF is transient and whether it is connected with successful decongestion.^7^ Indeed, some studies demonstrated that WRF does not adversely affect prognosis in effectively decongested patients.^27^ The incidence of WRF was similar between groups in this meta‐analysis. And the stability of renal function with advanced clinical benefit such as dyspnea relief could be regarded as an active finding. Since achieving similar effects just by using more loop diuretics might have increased the incidence of WRF as seen in DOSE (Diuretic Optimization Strategies Evaluation in Acute Heart Failure) study.^28^

This study met most of the methodological criteria suggested for systematic reviews and meta‐analyses.^29^ However, several limitations need to be considered in interpreting the results of the present study. First, some potential confounding between‐study variables could have influenced outcomes and thus may have also affected our meta‐analysis results. Second, the current meta‐analysis was not patient level and therefore results should be considered provisional.

## 5 CONCLUSIONS

The use of tolvaptan significantly improved patient‐assessed dyspnea early to 12 h, and persisted up to 72 h in patients with AHF. This meta‐analysis establishes that the addition of tolvaptan may be critical in early and persistently dyspnea relief in patients with AHF.

## CONFLICT OF INTEREST

The authors declare no conflict of interest.

## Data Availability

All data generated or analyzed during this study are included in this article. Further inquiries can be directed to the corresponding author.
